# On the choice of negative examples for prediction of host-pathogen protein interactions

**DOI:** 10.3389/fbinf.2022.1083292

**Published:** 2022-12-15

**Authors:** Don Neumann, Soumyadip Roy, Fayyaz Ul Amir Afsar Minhas, Asa Ben-Hur

**Affiliations:** ^1^ Department Computer Science, Colorado State University, Fort Collins, CO, United States; ^2^ Department of Computer Science, University of Warwick, Coventry, United Kingdom

**Keywords:** protein-protein interactions, host-pathogen interactions, deep learning, machine learning method evaluation, negative examples

## Abstract

As practitioners of machine learning in the area of bioinformatics we know that the quality of the results crucially depends on the quality of our labeled data. While there is a tendency to focus on the quality of positive examples, the negative examples are equally as important. In this opinion paper we revisit the problem of choosing negative examples for the task of predicting protein-protein interactions, either among proteins of a given species or for host-pathogen interactions and describe important issues that are prevalent in the current literature. The challenge in creating datasets for this task is the noisy nature of the experimentally derived interactions and the lack of information on non-interacting proteins. A standard approach is to choose random pairs of non-interacting proteins as negative examples. Since the interactomes of all species are only partially known, this leads to a very small percentage of false negatives. This is especially true for host-pathogen interactions. To address this perceived issue, some researchers have chosen to select negative examples as pairs of proteins whose sequence similarity to the positive examples is sufficiently low. This clearly reduces the chance for false negatives, but also makes the problem much easier than it really is, leading to over-optimistic accuracy estimates. We demonstrate the effect of this form of bias using a selection of recent protein interaction prediction methods of varying complexity, and urge researchers to pay attention to the details of generating their datasets for potential biases like this.

## 1 Introduction

Prediction of protein-protein interactions (PPIs), and more recently host-pathogen interactions (HPIs) is a very active area of research in computational biology (Lian et al., 2021; Hu et al., 2022a). Most of the work in this area focuses on prediction of interactions from sequence, especially using deep learning techniques. Some recent publications reported highly accurate prediction results *from sequence alone* that caught our attention (Tsukiyama et al., 2021; Asim et al., 2022; Madan et al., 2022). As long-time practiotioners of machine learning in this area, we approach such results with a healthy dose of skepticism. What could be the cause of such high accuracy? In this paper we focus on one issue related to the choice of negative examples that keeps showing up in various guises.

While databases of PPIs and HPIs are abundant and provide curated information on protein interactions, finding reliable examples of non-interacting proteins is more of a challenge. The Negatome database is one such resource (Blohm et al., 2014); however, the number of interactions in it is very limited and much smaller than the number of experimentally determined interactions, and does not cover HPIs. In the absence of gold-standard non-interacting proteins, some researchers have chosen to constrain their negative examples in various ways—either by protein localization, justified by the fact that proteins that reside in different cellular compartments are less likely to interact (Martin et al., 2005) or by constraining the similarity of negative examples to known positive examples (Eid et al., 2016). These approaches produce more reliable negative examples than the alternative of choosing random pairs of proteins that are not known to interact, reducing the number of false negatives. However, PPI networks are expected to be very sparse, and therefore the false negative rate for the random pairs method of choosing negative examples is expected to be very small Ben-Hur and Noble (2006). And as we have discussed elsewhere Ben-Hur and Noble (2006), the bias introduced by choosing negative examples according to their localization makes the problem easier, inflating prediction performance. Yet another way to introduce a bias on the choice of negative examples is to use proteins with low degrees in the interaction network, since these are less likely to interact with a viral protein of interest Dey et al. (2020).

Eid et al. (2016) suggested that while PPI networks are indeed sparse, HPI networks are less likely to be so. On the basis of this hypothesis they proposed to choose negative examples by constraining their similarity to positive examples. More specifically, if a host protein is part of the positive set, negative examples of similar host proteins are excluded, since they constitute potential interactions. As we describe below, this is a very effective way of making the prediction problem easier, and indeed provides improved performance. This was demonstrated by Eid et al. and shown here using current deep learning methods. However, this practice is wrong from a machine learning perspective, and we argue that its performance is not expected to hold for real data.

Although some researchers have rightfully shunned the technique of similarity-constrained negative example selection (Liu-Wei et al., 2021; Madan et al., 2022), this practice remains present in the field of HPI prediction (Basit et al., 2018; Zhou et al., 2018; Yang et al., 2020; Pitta et al., 2021; Tsukiyama et al., 2021; Yang et al., 2021; Asim et al., 2022) and also in PPI prediction Chen et al. (2022), necessitating this paper to alert researchers to this issue. We have also observed the use of similarity based choice of negative examples in other sequence-based prediction problems such as anti-microbial peptide prediction (Veltri et al., 2018). We note that the related practice of using cellular compartment to bias the choice of negative examples is also still occasionally being used (Sun et al., 2017). The very high accuracy reported in some of the publications cited above may create the wrong impression regarding the accuracy of predicting HPIs from sequence, and it is important that as method developers we be aware of all the potential pitfalls in designing our machine learning experiments.

## 2 Results and discussion

**FIGURE 1:**
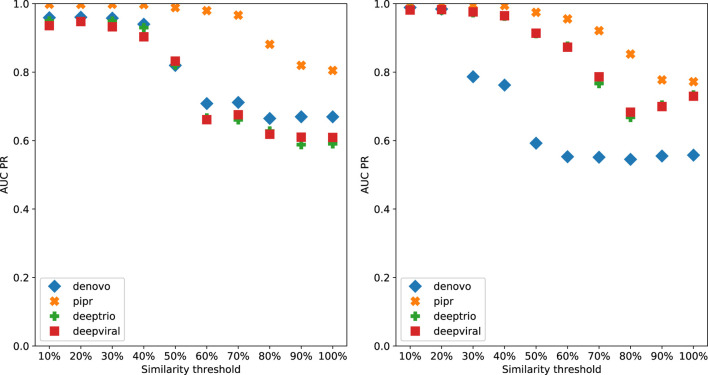
*Denovo* datasets with negative pathogen-host protein pairing by sequence similarity reported as AUCPR for each model, left: originally published *Denovo* datasets, right: HPIDB based *Denovo* datasets.

**FIGURE 2:**
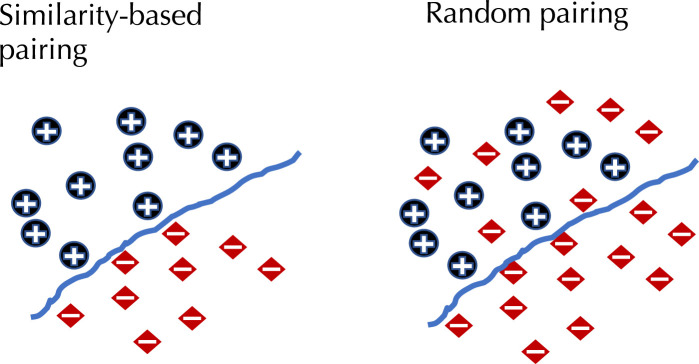
The effect of similarity-based selection of negative examples. When using similarity-based selection of negative examples this forces a distinction between positive and negative examples, making the problem much easier to solve.

To demonstrate the effect of using similarity-based sampling on HPI prediction accuracy we implemented the strategy proposed by Eid et al. (2016) and created training and test sets characterized by a threshold of the maximum allowed sequence similarity between host proteins that participate in the training and tests sets (see details in the Methods section). In addition to the original Support Vector Machine (SVM) model of Eid et al., we applied this strategy to a selection of deep learning models that were developed for PPI and HPI prediction. Model performance was assessed using five fold cross validation for varying sequence similarity thresholds for datasets constructed using two collections of positive examples: the dataset used in Eid et al., and a larger dataset generated using the latest version of the Host-Pathogen Interaction Database (HPIDB). Results are shown in Figure 1. The general trend for all the methods is that performance as measured by the area under the precision recall curve (AUPR) decreases as the similarity threshold increases. For low values of the similarity threshold, i.e. when the distinction between proteins in the training and test sets is extremely well pronounced all the methods achieve close to perfect accuracy, even the simple SVM-based method that uses trimer composition of the two proteins to represent the data. As the similarity threshold increases, the problem becomes more difficult as test set proteins are allowed to become more similar to proteins in the training set. In this regime, the SVM performs at a level that is not much better than a random classifier. The situation is described in Figure 2: for a high similarity threshold, the sampling produces what are essentially random pairs that are not known to interact, and the two classes can overlap. As the similarity threshold decreases, the two classes are pushed further apart, making the problem increasingly easy to solve. If this is done just on the training set as in (Lanchantin et al., 2021), this is appropriate; however, when done on examples on the test set, it makes the test set easy by construction, providing the user with a false sense of success. In-fact, in related work, we have shown that negative examples chosen by constraining sequence similarity does not generalize as well as random pairs for the problem of protein-compound interaction prediction based on an independent test set that uses negative examples chosen as pairs that have low binding affinity (Yaseen et al., 2022). Some authors choose to use similarity-constrained negative examples only in the training set (Lanchantin et al., 2021). This way of using similarity-constrained negative examples is not problematic, since there is no information leakage between the training and test sets. However, we suspect that the reduced label noise is not sufficient to compensate for the resulting difference in the distribution of training and test set, and would result in lower prediction accuracy.

It is worth noting that PIPR, which is the most sophisticated deep learning method among those tested is able to maintain a reasonable level of accuracy even for random pairing, and is the most responsive to even low deviations from random sampling. All the other methods required more help in terms of the separation between train and test sets in order to achieve high accuracy.

## 3 Conclusion

In this paper, we discussed pitfalls in the selection of negative examples for host-pathogen and protein-protein interactions. There are other issues that come into play when designing machine learning experiments in this domain. While our focus was on negative example selection, there are multiple issues that are relevant for the choice of positive examples as well: data from experimental methods such as yeast-two-hybrid are known to have a sizable fraction of false positives, and it is common practice to select positive examples by choosing interactions that have been assigned a high confidence score Hamp and Rost (2015). Another issue is whether to include in the test set interactions for host or pathogen proteins that are present in the training set: if a protein is present in the training set, either as a host or pathogen protein, the classifier is better able to make accurate predictions. So, a naive cross-validation procedure like we have used here provides accuracy estimates that may over-estimate performance if the user is interested in performance over proteins that were unseen by the classifier. This has been discussed by (Park and Marcotte, 2012; Hamp and Rost, 2015) in the context of protein-protein interactions. A common evaluation procedure in HPI prediction is to test the method on novel pathogens for which no data is present in the training set. This captures a likely use case where we wish to obtain potential interactions for an emerging pathogen whose interactions are yet to be studied in the lab. The final issue we would like to mention is class imbalance. Since host-pathogen interaction networks are expected to be sparse, the number of negative examples is expected to be much larger than the number of positive examples, leading to highly imbalanced classification problem. This has impact on the expected classification performance as demonstrated in a recent publication on PPI prediction (Dunham and Ganapathiraju, 2021). Unlike the area under the ROC curve which is invariant to class imbalance, more realistic measures like the area under the precision-recall curve are strongly affected by class imbalance. In summary, we call upon authors to be aware of these issues and exercise good experiment design that provides valid indication of the method’s performance in the real world.

## 4 Materials and methods

### 4.1 Models

The models we selected for our experiments cover a wide variety of sequence based published machine learning methods for HPI prediction from simple methods like the SVM from the original *Denovo* paper (Eid et al., 2016) and the single layer convolutional methods DeepViral Liu-Wei et al. (2021) and Hu et al. (2022a), to more complex methods like PIPR Chen et al. (2019). In our work we used the original *Denovo* SVM method (Eid et al., 2016) as a baseline. The model represents a pair of protein sequences in terms of their k-mer composition vectors normalized to unit vectors and concatenated, to which a Gaussian kernel is applied. Our implementation uses scikit-learn Pedregosa et al. (2011) SVM implementation after verifying it produced the same results on their original datasets, and uses 3-mers in a reduced amino-acid alphabet as in the original publication (Eid et al., 2016).

We also chose a selection of sequence-based deep learning methods of varying complexity. The simplest, DeepViral Liu-Wei et al. (2021), is a fully convolutional network which uses a single convolutional layer composed of eight different convolutional modules executed in parallel, with convolution applied independently to each protein and concatenated. In our implementation we removed the dropout on the convolutional layer, as we found the model performs much better without it. This is the sequence-only variant of DeepViral, for a fair comparison with the other methods. Each sequence is one hot encoded and the models were trained for 30 epochs.

PIPR Chen et al. (2019) is a more elaborate deep learning architecture for protein-protein interaction prediction comprised of multiple layers of convolution and gated recurrent units. PIPR encodes each amino acid using a vector that combines amino acid composition in a reduced seven dimensional space obtained by clustering amino acids by their properties Shen et al. (2007) with a set of features generated using the word2vec skip-gram model which represents the co-occurence of amino acids. The skip-gram model was trained on 8,000 sequences from the STRING protein-protein interaction network database Szklarczyk et al. (2016). We trained the models for 100 epochs as in the original publication.

We also used DeepTrio Hu et al. (2022b), a deep learning PPI prediction fully convolutional model which is comprised of 33 convolutional modules executed in parallel on the input sequence. The sequences are one hot encoded and the models were trained for 50 epochs.

All methods used a batch size of 256 with cross entropy loss, and were originally written in Keras and translated to PyTorch Paszke et al. (2019). Full implementations are provided on the github repository of this project at https://github.com/biodlab/hpi-neg.

### 4.2 Datasets

In our experiments we used datasets parameterized by the maximum allowed sequence similarity between host proteins in the train and test sets with thresholds ranging from 10% (highly constrained examples, allowing only up to 10% similarity) to 100% (no constraint on similarity between the host proteins in the train and test sets). The original *Denovo* dataset is comprised of 5,445 human-pathogen interactions, with 445 pathogen proteins and 2340 human protein derived from VirusMentha Calderone et al. (2015). These interactions were used to create 10 different datasets with similarity thresholds between 10% and 100%, where sequence similarity is computed using the Needleman-Wunsch algorithm Needleman and Wunsch (1970). For complete details of the algorithm we refer the reader to the original publication (Eid et al., 2016). In addition to the original *Denovo* dataset we created a second much larger dataset (Denovo-HPIDB) based on the latest Host-Pathogen Interaction Database (HPIDB) Ammari et al. (2016). HPIDB comprises multiple host and pathogen species, with human being the predominant host. All interactions were restricted to human host only which totaled 50,681 interactions between 9580 human proteins and 5930 pathogen proteins.

## Data Availability

The datasets presented in this study can be found in online repositories. The names of the repository/repositories and accession number(s) can be found below: https://github.com/biodlab/hpi-neg.

## References

[B1] Ammari M. G. Gresham C. R. McCarthy F. M. Nanduri B. (2016). HPIDB 2.0: A curated database for host–pathogen interactions. Oxford Academic. Database 2016. 10.1093/database/baw103 PMC4930832 27374121

[B2] Asim M. N. Ibrahim M. A. Malik M. I. Dengel A. Ahmed S. (2022). LGCA-VHPPI: A local-global residue context aware viral-host protein-protein interaction predictor. Plos one 17, e0270275. 10.1371/journal.pone.0270275 35789333 PMC9255777

[B3] Basit A. H. Abbasi W. A. Asif A. Gull S. Minhas F. U. A. A. (2018). Training host-pathogen protein–protein interaction predictors. J. Bioinform. Comput. Biol. 16, 1850014. 10.1142/s0219720018500142 30060698

[B4] Ben-Hur A. Noble W. S. (2006). Choosing negative examples for the prediction of protein-protein interactions. BMC Bioinforma. 7, S2–S6. 10.1186/1471-2105-7-s1-s2 PMC1810313 16723005

[B5] Blohm P. Frishman G. Smialowski P. Goebels F. Wachinger B. Ruepp A. (2014). Negatome 2.0: A database of non-interacting proteins derived by literature mining, manual annotation and protein structure analysis. Nucleic Acids Res. 42, D396–D400. 10.1093/nar/gkt1079 24214996 PMC3965096

[B6] Calderone A. Licata L. Cesareni G. (2015). VirusMentha: A new resource for virus-host protein interactions. Nucleic acids Res. 43, D588–D592. 10.1093/nar/gku830 25217587 PMC4384001

[B7] Chen M. Ju C. J. T. Zhou G. Chen X. Zhang T. Chang K. W. (2019). Multifaceted protein–protein interaction prediction based on siamese residual RCNN. Bioinformatics 35, i305–i314. 10.1093/bioinformatics/btz328 31510705 PMC6681469

[B8] Chen W. Wang S. Song T. Li X. Han P. Gao C. (2022). DCSE: Double-channel-siamese-ensemble model for protein protein interaction prediction. BMC genomics 23, 555–614. 10.1186/s12864-022-08772-6 35922751 PMC9351149

[B9] Dey L. Chakraborty S. Mukhopadhyay A. (2020). Machine learning techniques for sequence-based prediction of viral–host interactions between SARS-CoV-2 and human proteins. Biomed. J. 43, 438–450. 10.1016/j.bj.2020.08.003 33036956 PMC7470713

[B10] Dunham B. Ganapathiraju M. K. (2021). Benchmark evaluation of protein–protein interaction prediction algorithms. Molecules 27, 41. 10.3390/molecules27010041 35011283 PMC8746451

[B11] Eid F. E. ElHefnawi M. Heath L. S. (2016). DeNovo: Virus-host sequence-based protein–protein interaction prediction. Bioinformatics 32, 1144–1150. 10.1093/bioinformatics/btv737 26677965

[B12] Hamp T. Rost B. (2015). Evolutionary profiles improve protein–protein interaction prediction from sequence. Bioinformatics 31, 1945–1950. 10.1093/bioinformatics/btv077 25657331

[B13] Hu X. Feng C. Ling T. Chen M. (2022a). Deep learning frameworks for protein-protein interaction prediction. Comput. Struct. Biotechnol. J. 20, 3223–3233. 10.1016/j.csbj.2022.06.025 35832624 PMC9249595

[B14] Hu X. Feng C. Zhou Y. Harrison A. Chen M. (2022b). DeepTrio: A ternary prediction system for protein–protein interaction using mask multiple parallel convolutional neural networks. Bioinformatics 38, 694–702. 10.1093/bioinformatics/btab737 PMC8756175 34694333

[B15] Lanchantin J. Weingarten T. Sekhon A. Miller C. Qi Y. (2021). Transfer learning for predicting virus-host protein interactions for novel virus sequences. Proc. 12th ACM Conf. Bioinforma. Comput. Biol. Health Inf., 1–10.

[B16] Lian X. Yang X. Yang S. Zhang Z. (2021). Current status and future perspectives of computational studies on human–virus protein–protein interactions. Brief. Bioinform. 22, bbab029. 10.1093/bib/bbab029 33693490

[B17] Liu-Wei W. Kafkas Ş. Chen J. Dimonaco N. J. Tegnér J. Hoehndorf R. (2021). DeepViral: Prediction of novel virus–host interactions from protein sequences and infectious disease phenotypes. Bioinformatics 37, 2722–2729. 10.1093/bioinformatics/btab147 33682875 PMC8428617

[B18] Madan S. Demina V. Stapf M. Ernst O. Fröhlich H. (2022). Accurate prediction of virus-host protein-protein interactions via a siamese neural network using deep protein sequence embeddings. Patterns (N Y). 3 (9), 100551. 10.1016/j.patter.2022.100551 PMC9481957 36124304

[B19] Martin S. Roe D. Faulon J. L. (2005). Predicting protein–protein interactions using signature products. Bioinformatics 21, 218–226. 10.1093/bioinformatics/bth483 15319262

[B20] Needleman S. B. Wunsch C. D. (1970). A general method applicable to the search for similarities in the amino acid sequence of two proteins. J. Mol. Biol. 48, 443–453. 10.1016/0022-2836(70)90057-4 5420325

[B21] Park Y. Marcotte E. M. (2012). Flaws in evaluation schemes for pair-input computational predictions. Nat. Methods 9, 1134–1136. 10.1038/nmeth.2259 23223166 PMC3531800

[B22] Paszke A. Gross S. Massa F. Lerer A. Bradbury J. Chanan G. (2019). PyTorch: An imperative style, high-performance deep learning library. Adv. neural Inf. Process. Syst. 32.

[B23] Pedregosa F. Varoquaux G. Gramfort A. Michel V. Thirion B. Grisel O. (2011). Scikit-learn: Machine learning in Python. J. Mach. Learn. Res. 12, 2825–2830.

[B24] Pitta J. LdL. P. dos Santos Vasconcelos C. R. da Luz Wallau G. de Lima Campos T. Rezende A. M. (2021). *In silico* predictions of protein interactions between zika virus and human host. PeerJ 9, e11770. 10.7717/peerj.11770 34513323 PMC8395582

[B25] Shen J. Zhang J. Luo X. Zhu W. Yu K. Chen K. (2007). Predicting protein–protein interactions based only on sequences information. Proc. Natl. Acad. Sci. U. S. A. 104, 4337–4341. 10.1073/pnas.0607879104 17360525 PMC1838603

[B26] Sun T. Zhou B. Lai L. Pei J. (2017). Sequence-based prediction of protein protein interaction using a deep-learning algorithm. BMC Bioinforma. 18, 277–278. 10.1186/s12859-017-1700-2 PMC5445391 28545462

[B27] Szklarczyk D. Morris J. H. Cook H. Kuhn M. Wyder S. Simonovic M. (2016). The STRING database in 2017: Quality-controlled protein–protein association networks, made broadly accessible. Nucleic acids Res., gkw937. 10.1093/nar/gkw937 PMC5210637 27924014

[B28] Tsukiyama S. Hasan M. M. Fujii S. Kurata H. (2021). LSTM-PHV: Prediction of human-virus protein–protein interactions by LSTM with word2vec. Brief. Bioinform. 22, bbab228. 10.1093/bib/bbab228 34160596 PMC8574953

[B29] Veltri D. Kamath U. Shehu A. (2018). Deep learning improves antimicrobial peptide recognition. Bioinformatics 34, 2740–2747. 10.1093/bioinformatics/bty179 29590297 PMC6084614

[B30] Yang X. Yang S. Li Q. Wuchty S. Zhang Z. (2020). Prediction of human-virus protein-protein interactions through a sequence embedding-based machine learning method. Comput. Struct. Biotechnol. J. 18, 153–161. 10.1016/j.csbj.2019.12.005 31969974 PMC6961065

[B31] Yang X. Yang S. Lian X. Wuchty S. Zhang Z. (2021). Transfer learning via multi-scale convolutional neural layers for human–virus protein–protein interaction prediction. Bioinformatics 37, 4771–4778. 10.1093/bioinformatics/btab533 PMC8406877 34273146

[B32] Yaseen A. Amin I. Akhter N. Ben-Hur A. Minhas F. (2022). Insights into performance evaluation of compound–protein interaction prediction methods. Bioinformatics 38, ii75–ii81. 10.1093/bioinformatics/btac496 36124806

[B33] Zhou X. Park B. Choi D. Han K. (2018). A generalized approach to predicting protein-protein interactions between virus and host. BMC genomics 19, 568–577. 10.1186/s12864-018-4924-2 30367586 PMC6101077

